# Integrated analysis of single-cell and bulk RNA-sequencing reveals a novel signature based on NK cell marker genes to predict prognosis and immunotherapy response in gastric cancer

**DOI:** 10.1038/s41598-024-57714-7

**Published:** 2024-04-01

**Authors:** Jian-Rong Sun, Chen-Fan Kong, Yi-Xiang Ye, Qin Wang, Xiang-Ke Qu, Li-Qun Jia, Song Wu

**Affiliations:** 1 School of Clinical Medicine, Beijing University of Chinese Medicine, No. 11, North 3rd East Road, Beijing, 100029 Chaoyang People’s Republic of China; 2 Department of Urology, The affiliated Shenzhen Hospital of Shanghai University of Traditional Chinese Medicine, No. 16, Liantangxiantong Road, Shenzhen, 518009 Luohu People’s Republic of China; 3 Department of Urology, South China Hospital, Health Science Center, Shenzhen University, Shenzhen, 518116 People’s Republic of China

**Keywords:** Single-cell RNA-sequencing, Bulk RNA sequencing, NK cell, Prognostic signature, Tumor microenvironment, Immunotherapy response, Gastric cancer, Cancer, Computational biology and bioinformatics, Biomarkers

## Abstract

Natural killer (NK) cells play essential roles in the tumor development, diagnosis, and prognosis of tumors. In this study, we aimed to establish a reliable signature based on marker genes in NK cells, thus providing a new perspective for assessing immunotherapy and the prognosis of patients with gastric cancer (GC). We analyzed a total of 1560 samples retrieved from the public database. We performed a comprehensive analysis of single-cell RNA-sequencing (scRNA-seq) data of gastric cancer and identified 377 marker genes for NK cells. By performing Cox regression analysis, we established a 12-gene NK cell-associated signature (NKCAS) for the Cancer Genome Atlas (TCGA) cohort, that assigned GC patients into a low-risk group (LRG) or a high-risk group (HRG). In the TCGA cohort, the areas under curve (AUC) value were 0.73, 0.81, and 0.80 at 1, 3, and 5 years. External validation of the predictive ability for the signature was then validated in the Gene Expression Omnibus (GEO) cohorts (GSE84437). The expression levels of signature genes were measured and validated in GC cell lines by real-time PCR. Moreover, NKCAS was identified as an independent prognostic factor by multivariate analysis. We combined this with a variety of clinicopathological characteristics (age, M stage, and tumor grade) to construct a nomogram to predict the survival outcomes of patients. Moreover, the LRG showed higher immune cell infiltration, especially CD8+ T cells and NK cells. The risk score was negatively associated with inflammatory activities. Importantly, analysis of the independent immunotherapy cohort showed that the LRG had a better prognosis and immunotherapy response when compared with the HRG. The identification of NK cell marker genes in this study suggests potential therapeutic targets. Additionally, the developed predictive signatures and nomograms may aid in the clinical management of GC.

## Introduction

Gastric cancer (GC) is the fifth most common malignancy worldwide, with 1,089,103 newly reported cases and 768,793 newly reported deaths in 2020^1^. Most patients are diagnosed with advanced-stage GC due to its insidious onset and the lack of overt early symptoms, thus resulting in missed opportunities for surgical resection. Despite recent improvements in the development of treatment approaches for advanced GC, the 5-year survival rate of less than 20% is still considered unsatisfactory^2^. Over the past decade, anti-PD-1/PD-L1 immune checkpoint blockade (ICB) therapy has revolutionized the treatment of many types of cancer. However, due to the heterogeneity of tumors, the overall response rate of ICB is relatively low and only a subset of GC patients could derive clinical benefit from such therapy^3,4^. Given this, it is necessary to identify novel biomarkers to predict the prognosis and immunotherapy response of GC.

The tumor microenvironment (TME) refers to the non-cancerous cells and components that are presented in a tumor, including the molecules produced and released by these cells and components. The constant interactions between tumor cells and the TME play decisive roles in tumor initiation, progression, metastasis, and response to therapies^5^. An increasing body of data suggests that the abundance and diversity of immune cells infiltrating the TME can significantly affect both the efficacy of immunotherapy and tumor growth^6^. Most current treatment options that harness the TME focus on T cell-immunity; however, the limited success of this technique highlights the importance of developing new-generation immunotherapies.

NK cells are a population of innate lymphoid cells that play a pivotal role in host immune responses against infection and tumor growth^7^. A previous study suggested that the low activity of NK cells in the peripheral blood is associated with an increased risk of cancer^8^. The higher abundance of infiltrating NK cells in the TME correlates with a favorable prognosis of some malignancies^9–11^. Furthermore, NK cell therapy can effectively improve the outcome of oncology treatment, thus presenting us with a promising perspective for cancer immunotherapy^12,13^. The molecular characteristics of NK cells have been reported in several solid tumors; however, the precise function of NK cells in GC remains unclear in terms of diagnosis and prognosis^14–16^.

Single-cell RNA-sequencing (scRNA-seq) technology enables the comprehensive characterization of the cellular compositions and transcriptional phenotypes in the TME. Previous studies have reported that a gene expression signature based on immune cells derived from scRNA-seq data could potentially be a powerful method for predicting the prognosis and response to immunotherapy of cancer patients^14,17,18^. In the present study, we investigated the molecular characteristics of the GC microenvironment by analyzing scRNA-seq data and identifying specific marker genes for NK cells. Then we established and validated a NK cell-associated signature (NKCAS) to predict prognosis through bulk RNA-seq analysis. Moreover, we investigated the immune microenvironment and the relationship between the NKCAS and immunotherapy response in GC patients.

## Methods

### Data collection

A total of 1560 samples were included in the current study, namely, 8 samples with scRNA-seq data of GC from the Gene Expression Omnibus (GEO, GSE183904), 371 samples of GC from the Cancer Genome Atlas (TCGA), 833 samples of GC from the Gene Expression Omnibus (GEO, 433 samples from GSE84437 and 400 samples from GSE66229) (Supplementary Table 1), and 348 samples of urothelial carcinoma receiving immunotherapy from the IMvigor 210 cohort (Supplementary Table 2). The scRNA-seq dataset was used to determine the signature genes for NK cells in GC. The bulk RNA-seq data and corresponding clinical annotations of GC patients were acquired from the TCGA to identify genes associated with prognosis and establish a predictive signature. The independent microarray datasets (GEO, GSE84437) were used to perform the external verification of the signature for predicting the survival outcomes. Another independent dataset (GEO, GSE66229) was used to validate the expression of the signature genes. The transcriptomic profile and corresponding clinical information of the IMvigor210 dataset (in which patients of urothelial carcinoma received anti-PD-L1 treatment) were used to investigate the speculative value of a NK cell-associated signature (NKCAS) on immunotherapy response. Particularly, due to the scarcity of immunotherapy cohorts for GC, we investigated the ability of the NKCAS to predict immunotherapy response using the IMvigor210 cohort, which has been widely used in other cancer types.

### Identification of NK cell signature genes by scRNA-seq analysis

In the scRNA-seq (GSE183904) cohort, the “Seurat” and “Single R” tools in R software were used to conduct scRNA-seq data analysis. First, we removed the clusters with cell counts < 3. Cells with < 50 mapped genes and cells in which mitochondrial genes exceeded 5% were also removed. Then, we performed data normalization by utilizing the “NormalizeData” package in R software. The top 15 principal components were selected for principal component analysis (PCA) based on the top 1500 variably expressed genes. T-distributed stochastic neighbor embedding (t-SNE) was used for unsupervised clustering and to visualize cell subpopulations on a two-dimensional map in a non-biased manner. The Human Primary Cell Atlas Data was used to annotate the cell clusters. The “FindAllMarkers” function in R software was then used to compare differences in gene expression among clusters. Genes with a |log2 (fold change) |> 1 and an adjusted *P*-value < 0.05 were regarded as signature genes. Finally, we used the “SingleR” tool in the R package to annotate cell subpopulations from the different clusters.

### Establishment and verification of the NK cell-associated signature (NKCAS)

In the TCGA cohort, we used univariate Cox regression analysis to evaluate the predictive ability of NK cell signature genes on the survival of GC patients. Prognostic genes were identified with a significance level of *P* < 0.05. Then, to avoid overfitting and enhance the robustness of the prognostic signature, we performed the least absolute shrinkage and selection operator (LASSO) Cox proportional hazards regression analysis to select optimal prognostic genes. Then ten-fold cross-validation was used to select the ideal model, and the tuning parameter λwas chosen by 1-SE (standard error). Finally, based on the genes screened by LASSO Cox regression analysis, we conducted the multivariate Cox regression analysis to generate a prognostic signature. The formula used to calculate the NKCAS risk score was as follows:

$${\text{Risk Score}} = \mathop \sum \limits_{{{\text{k}} = 1}}^{{\text{n}}} {\text{Coef}}_{{\text{k}}} \times {\text{B}}_{{\text{k}}}$$

in which ${\text{Coef}}_{{\text{k}}}$ represented the coefficient and ${\text{B}}_{{\text{k}}}$ represented the normalized expression value of the NK cell signature genes. Each patient was given a risk score based on the formula and then all patients were assigned into a low-risk group (LRG) or a high-risk group (HRG) by the median value of the risk score. Receiver operating characteristic (ROC) curves were plotted and the area under curve (AUC) was calculated by the “survivalROC” package in R; AUC values were then used to evaluate the predictive efficacy of the NKCAS model. The Kaplan–Meier (KM) curves and log-rank (LR) tests were conducted to determine the differences of overall survival (OS) between the two risk groups. The robustness of the NKCAS model was then validated in the independent GEO datasets (GSE84437).

### Exploration of the mRNA expression levels of signature genes

Next, we compared the mRNA expression levels of signature genes between GC tumor samples and normal samples derived from the TCGA database. The GSE66229 dataset from the GEO database was used to validate the results of the signature genes involved in the prognostic model.

### Validation of the prognostic signature by relative quantitative real-time PCR (qPCR) and immunofluorescence

The expression levels of signature genes were measured in three GC cell lines (AGS and MKN-45, human gastric cells) and a control cell line (GES-1, human gastric mucosal epithelial cells). All the cell lines were obtained from the National Infrastructure of Cell Line Resources (Beijing, China) and were in RPMI-1640 (Gibco, USA), 10 fetal bovine serum (FBS, Gibco, USA), and 1% penicillin/streptomycin (Gibco, Canada). All the cells were cultured at 37℃ with 5% CO2. Total RNA was extracted from cells using the RNAsimple Total RNA Kit (TIANGEN, China, Cat. 4992858), and reverse transcription was subsequently performed using the FastKing gNDA Dispelling RT SuperMix (TIANGEN, China, Cat. 4992227). qPCR was performed with a SYBR Green Real-time PCR Kit (TIANGEN, China, Cat. 4992881) on a QuantStudio 5 Real-Time PCR System (Thermo Fisher Scientific, USA). All experiments were repeated at least three times. The RNA primer sequences are listed in Supplementary Table 3, Relative expression was calculated using the comparative threshold cycle (Ct) method.

Furthermore, we validated the level of proteins encoded by these signature genes in GC cell line (AGS) and control cell line (GES-1) via immunofluorescence. The antibodies for immunofluorescence including CXCR4 (Cat# ab181020), SHOX2 (Cat# ab55740), MSI2 (Cat# ab76148) and PLCL1 (Cat# ab157200) were purchased from Abcam (USA). RDH8 (Cat# PA5-139867), GRB14 (Cat# PA5-101612), SLC35E4 (Cat# PA5-62009), NEK5 (Cat# PA5-101860) and AKAP5 (Cat# PA5-101095) were purchased form Thermo (USA). MAGEA11(Cat# 15474-1-ap), CYP191A (Cat# 16554-1-ap) and KYNU (Cat# 11796-1-ap) were purchased from Proteintech (China). Cy3 conjugated Donkey Anti-Mouse IgG (Cat# GB21401), Cy3 conjugated Donkey Anti-Rabbit IgG (Cat# GB21403), FITC conjugated Donkey Anti-Rabbit IgG (Cat# GB22403), FITC conjugated Donkey Anti-Mouse IgG (Cat# GB22401) and Cy5 conjugated Goat Anti-rabbit IgG (Cat# GB27303) were purchased from Servicebio (China). The cells were treated with 4% paraformaldehyde and 0.1% Triton X-100 for fixation and penetration. After blocked with serum, cells were incubated with primary antibodies at room temperature for 1.5 h. Then cells were incubated with secondary antibody for 1 h. Finally, the nuclei were stained with DAPI.

### Development and efficiency of a NKCAS-clinicopathologic nomogram

To facilitate clinical application and provide a more convenient tool for predicting the prognosis of GC patients, we established a nomogram was established based on the NKCAS and clinical parameters.

The nomogram consists of four main sections: points, variables, total points, and 1,3,5-year survival rate. The point corresponding to each variable for a given patient is summed to the total point, and the total points draws a vertical line down corresponding to the patient’s 1,3,5-year survival rate. Multivariate Cox regression analysis was performed to identify the independent prognostic factors and then to establish a nomogram. ROC curves and time-dependent AUC values were used to evaluate predictive ability. Calibration curves were adopted to evaluate the consistency between prediction and actual values with a diagonal line indicating the first-rank prediction. Finally, decision curve analysis (DCA) was used to appraise the clinical applicability.

### Gene mutation landscape

Somatic mutation data from GC patients were acquired from the TCGA. The somatic mutation characteristics between different risk groups were then analyzed by the “maftools” package in R software. Furthermore, the tumor mutation burden (TMB) for each patient was calculated as mutations per million bases, and then all GC patients were split into low- or high-TMB groups according to TMB median value to investigate their impact on survival. In addition, we evaluated the impact of TMB together with risk stratification on survival.

### Landscape of the tumor immune microenvironment in GC

The immune score, stromal score, and ESTIMATE score of GC patients, as calculated by the ESTIMATE algorithm, were used to estimate the abundance of stromal cells and infiltrating immune cells in malignant tumors to predict the purity of tumors. In addition, the CIBERSORT algorithm was performed to evaluate the proportions of 22 immune cell types in each GC tumor sample. The H&E staining of TCGA pathology slides from different risk groups was used to detect the immune infiltrating cells in TME, to confirm the aforementioned analysis. Moreover, a seven-metagene (*HCK*, *IgG*, Interferon, *LCK*, *MHC-I, MHC-II*, and *STAT1*), representing the diverse inflammatory and immune activities that were described previously in the literature, was used to compare inflammatory activities between the LRG and HRG^19^. In addition, a heatmap was plotted by the “pheatmap” package in R to visualize discrepancies between the two groups. Spearman analysis was performed to examine the relationship between the risk scores and the seven metagenes. A correlation pie chart of metagenes and risk scores was then plotted by the “corrgram” package in R. Red represents a positive correlation, blue represents a negative correlation, and a larger pie chart represents a stronger correlation.

### Immunotherapy response prediction

Next, the tumor immune dysfunction and exclusion (TIDE) score, PD-L1 expression, and TMB were analyzed to predict the response to ICB therapy. The TIDE score was calculated from an online website (http://tide.dfci.harvard.edu/). The PD-L1 mRNA expression data from patients with GC were downloaded from the TCGA cohort. A gene mutation profile was acquired from the TCGA and the TMB was computed as mutations per million bases. In addition, the immunotherapy cohort (IMvigor210), featuring both transcriptomic data and treatment response, was used to verify the value of the NKCAS for predicting immunotherapeutic response.

### Ethics statement

As our data were downloaded from the online public database, there is no requirement for ethics committee approval and consent to participate.

## Results

### Identification of NK cell signature genes

**Figure 1:**
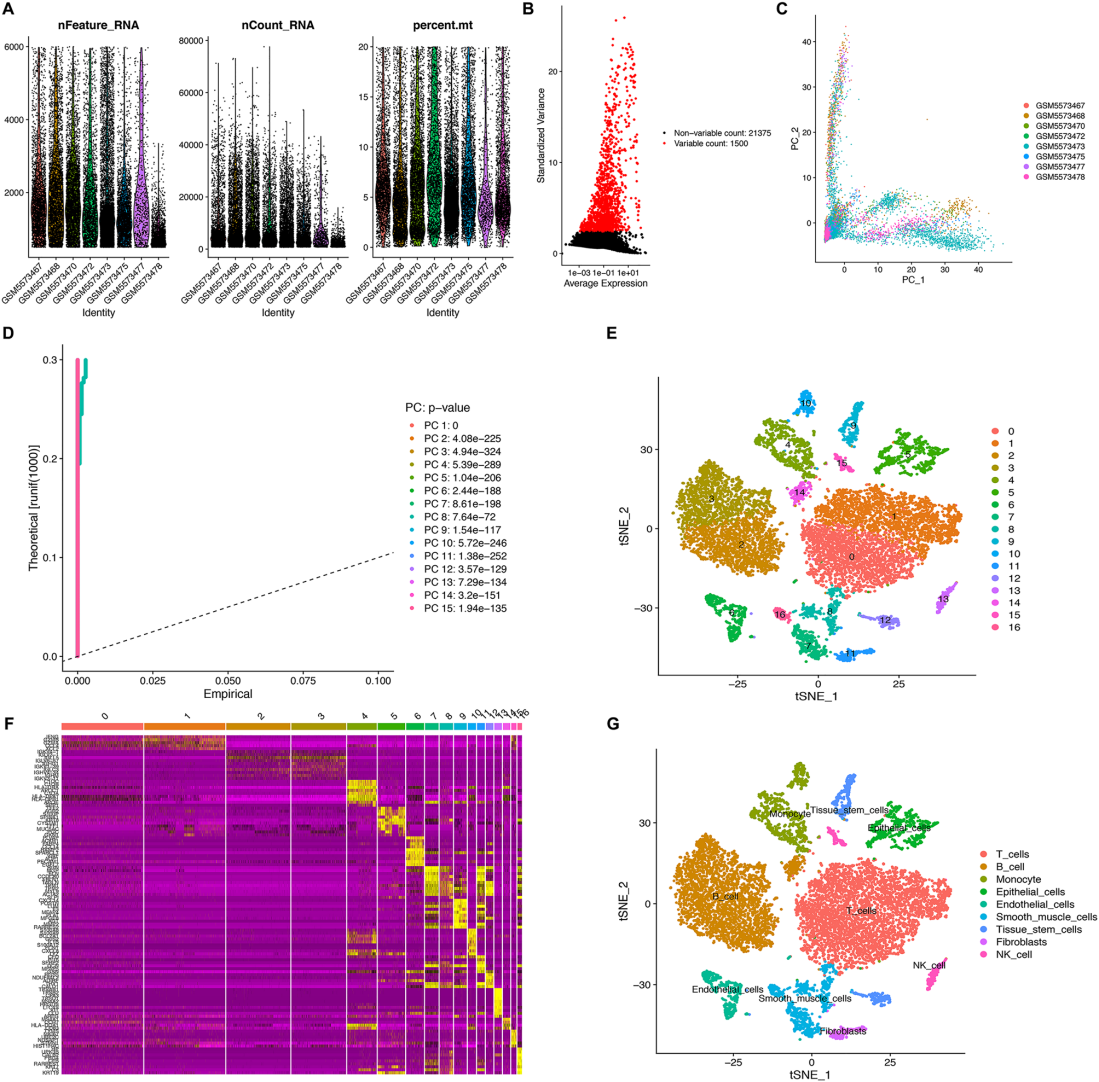
scRNA-seq analysis identifies NK cell marker genes. (**A**) Quality control of scRNA-seq data from eight GC samples. (**B**) The variance plot showed 1500 genes in all cells, red dots represent the top 1500 highly variable genes. (**C**) PCA was utilized for dimensionality reduction. (**D**) 15 PCs were identified based on *P*-value < 0.05. (**E**) t-SNE plot colored by various cell clusters. (**F**) Heatmap showing the top marker genes in each cell cluster. (**H**) The cell subpopulations identified by marker genes.

The gene expression profiles of 24,860 cells from eight primary GC samples were acquired from the scRNA-seq dataset (GSE183904). After data normalization, the top 1500 variable genes were selected and used for PCA analysis to reduce the dimensionality (Fig. 1A–C), and 15 PCs with *P* < 0.05 were chosen for further analysis (Fig. 1D). We conducted t-SNE analysis to visualize cell distribution and then identified 17 cell clusters, and each cluster showed distinct gene expression profiles (Fig. 1E–F). We used the “singleR” algorithm to annotate different cell subpopulations and identified cluster 13 as a subpopulation of NK cells (Fig. 1G). Finally, a total of 377 genes were identified in the NK cell cluster; these were considered as NK cell signature genes (Supplementary Table 4).

### Establishment and validation of a NKCAS for the prognostic assessment of GC

**Figure 2:**
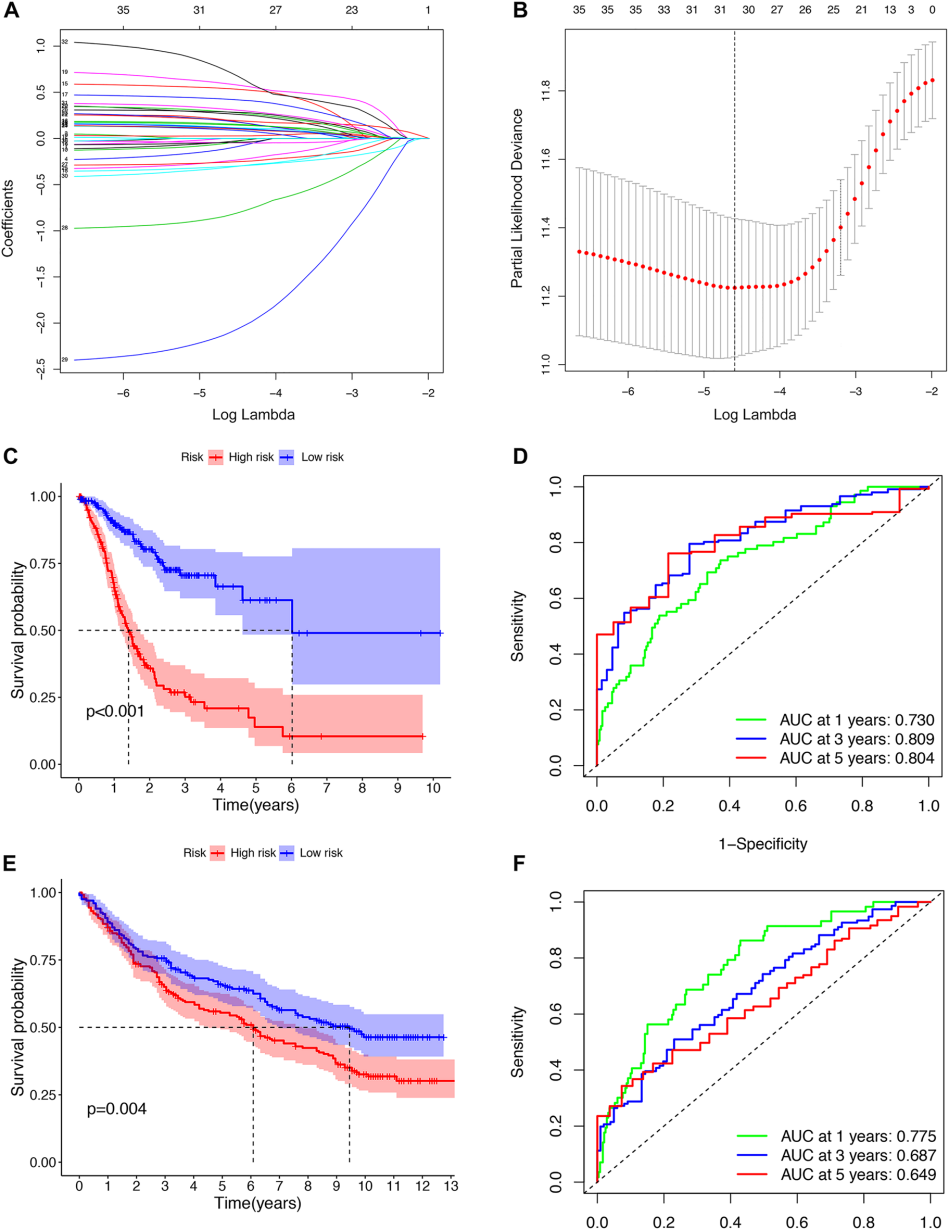
Construction and validation of the NK cell-associated signature (NKCAS). (**A**–**B**) LASSO regression analysis. (**C**) The Kaplan–Meier curves in the TCGA cohort. (**D**) The AUCs at 1-, 3-, and 5-year of the NKCAS in TCGA cohort. (**E**) The Kaplan–Meier curves in the GEO cohort (GSE84437). (F) The AUCs at 1-, 3-, and 5-year of the NKCAS in GEO cohort (GSE84437).

The TCGA cohort was then used to perform the univariate Cox regression analysis, and a total of 36 genes were found to be associated with the OS of GC patients (Supplementary Fig. 1). LASSO analysis identified 25 genes based on the optimal lambda value (one standard error) and tenfold cross-validation (Fig. 2A–B, Supplementary Table 5). Finally, multivariate Cox regression analysis was conducted to establish an optimal prognostic signature based on the 12 most predictive NK cell signature genes (*CXCR4, RDH8, MAGEA11, CYP19A1, SHOX2, GRB14, SLC35E4, NEK5, AKAP5, MSI2, KYNU,* and *PLCL1*). Based on their coefficients of these genes, the risk score formula was computed as follows:

Risk score = (0.247 × *CXCR4*expression) + (0.589 × *RDH8*expression) + (0.477 × *MAGEA11*expression) + (0.916 × *CYP19A1*expression) + (0.480 × *SHOX2*expression) + (0.400 × *GRB14*expression) + ( − 0.439 × *SLC35E4*expression) + (− 1.04 × *NEK5*expression) + (− 2.11 × *AKAP5*expression) + ( − 0.44 × *MSI2*expression) + (0.377 × *KYNU*expression) + (0.868 × *PLCL1*expression). The GC patients were assigned into the HRG (n = 185) and LRG (n = 186) groups according to the median risk score (1.042). The KM analysis indicated that the LRG showed significant better survival when compared with the HRG (Fig. 2C). The AUCs of the training set at 1-, 3-, and 5-years were 0.730, 0.809, and 0.804, respectively (Fig. 2D). To validate the predictive efficacy of the signature, the same analysis was conducted in the GEO dataset. Analysis showed that survival was better in the LRG than in the HRG (Fig. 2E). The AUCs of the GEO cohort at 1-, 3-, and 5-years were 0.775, 0.687, and 0.649, respectively (Fig. 2F). A scatter plot of risk score and survival status in both the TCGA and GEO cohorts revealed a higher mortality rate in the HRG (Supplementary Fig. 2A–B). Detailed expression data for the 12 NK cell signature genes are presented in Supplementary Fig. 2C–D. These results suggested that the NKCAS could maintain it predictive ability in different cohorts of patients.

### Differential expressions of the signature genes

**Figure 3:**
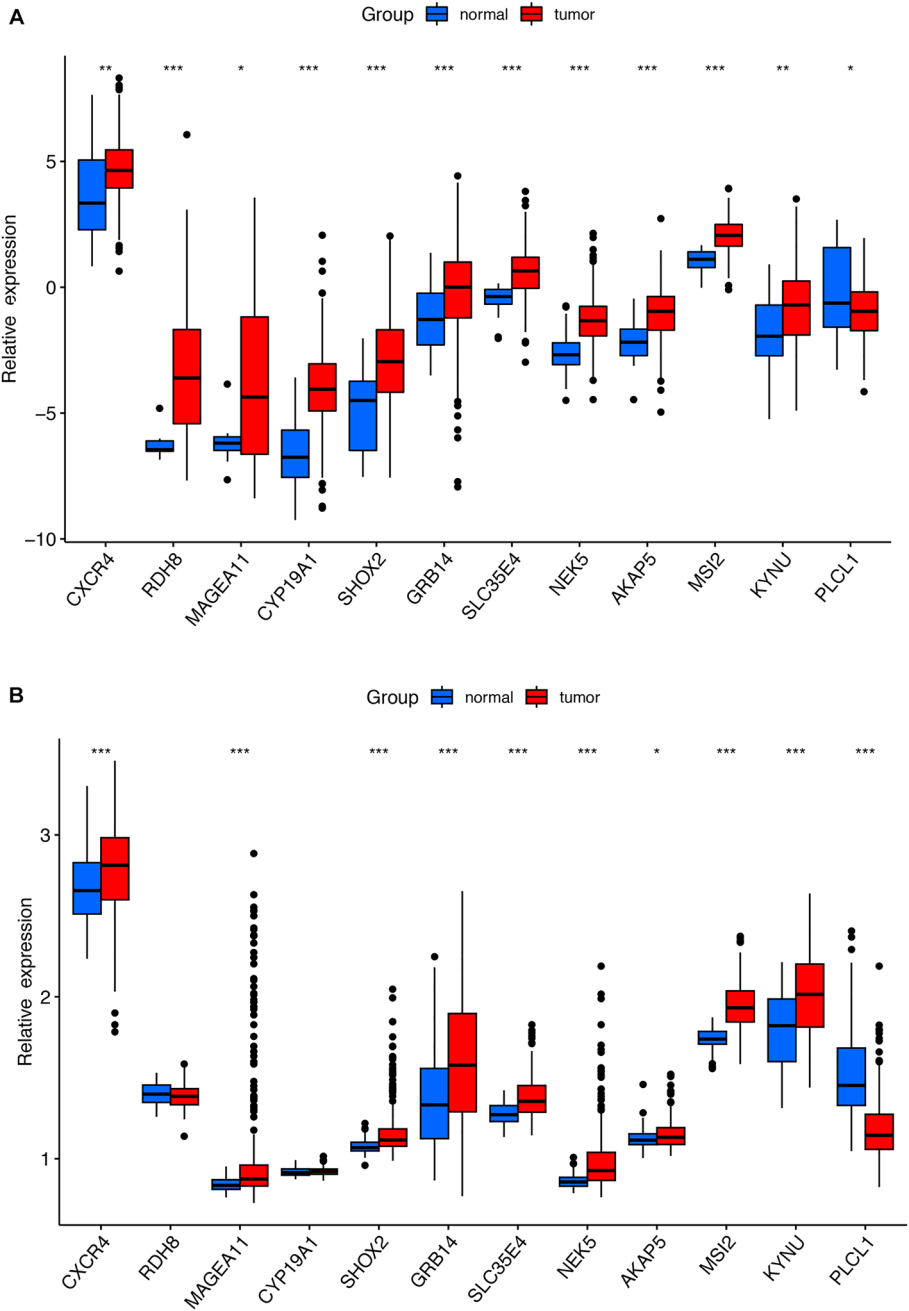
The expression of signature genes in TCGA and GEO datasets. (**A**) The expression of signature genes in TCGA datasets. (**B**) The expression of signature genes in GSE66229.

**Figure 4:**
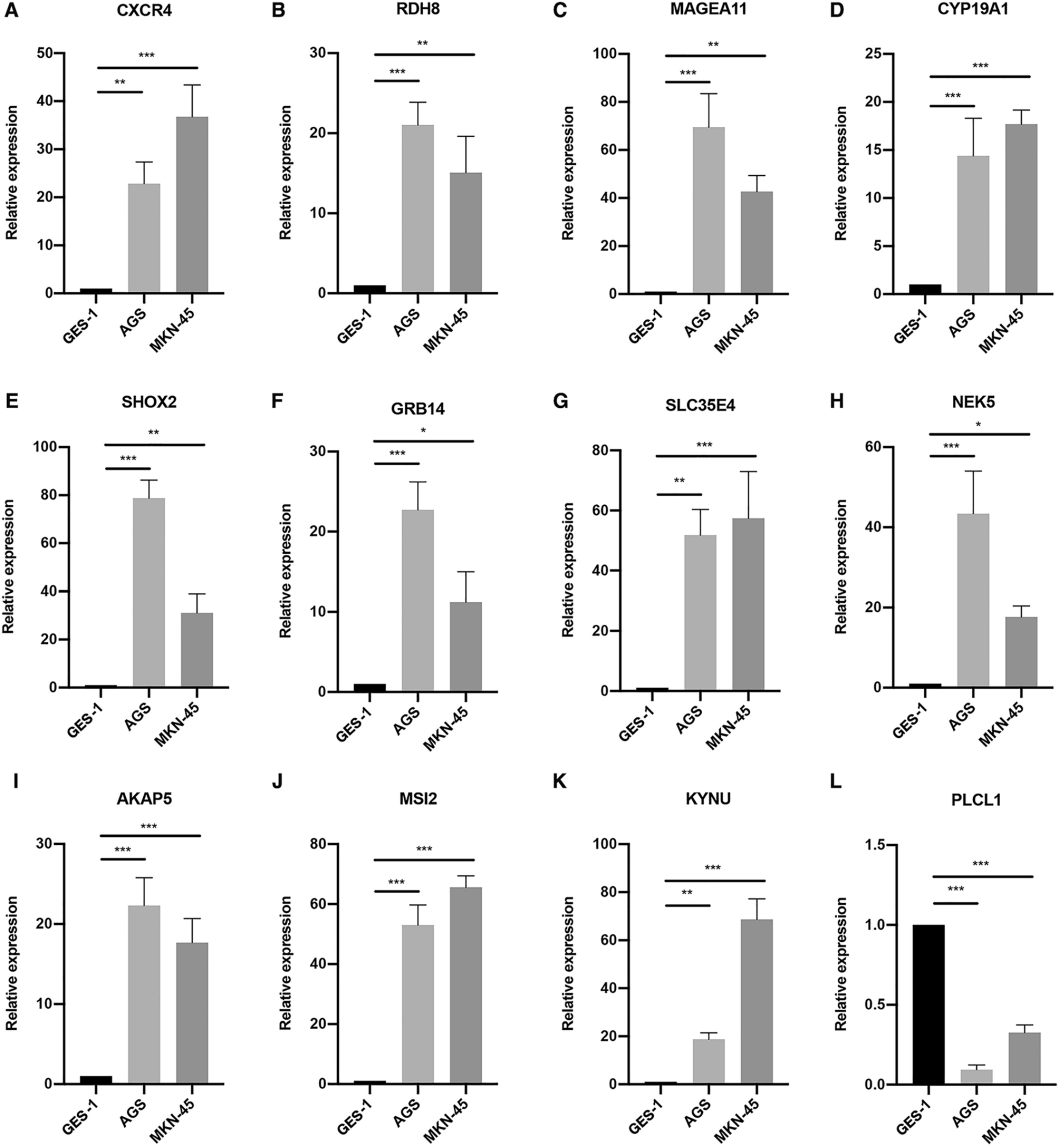
The expression of prognostic signature in cell lines. (**A**–**L**) The qPCR results of CXCR4, RDH8, MAGEA11, CYP19A1, SHOX2, GRB14, SLC35E4, NEK5, AKAP5, MSI2, KYNU, and PLCL1 in GC cell lines (AGS, and MKN45) and control cell lines (GSE-1). *P < 0.05, **P < 0.01, ***P < 0.001.

**Figure 5:**
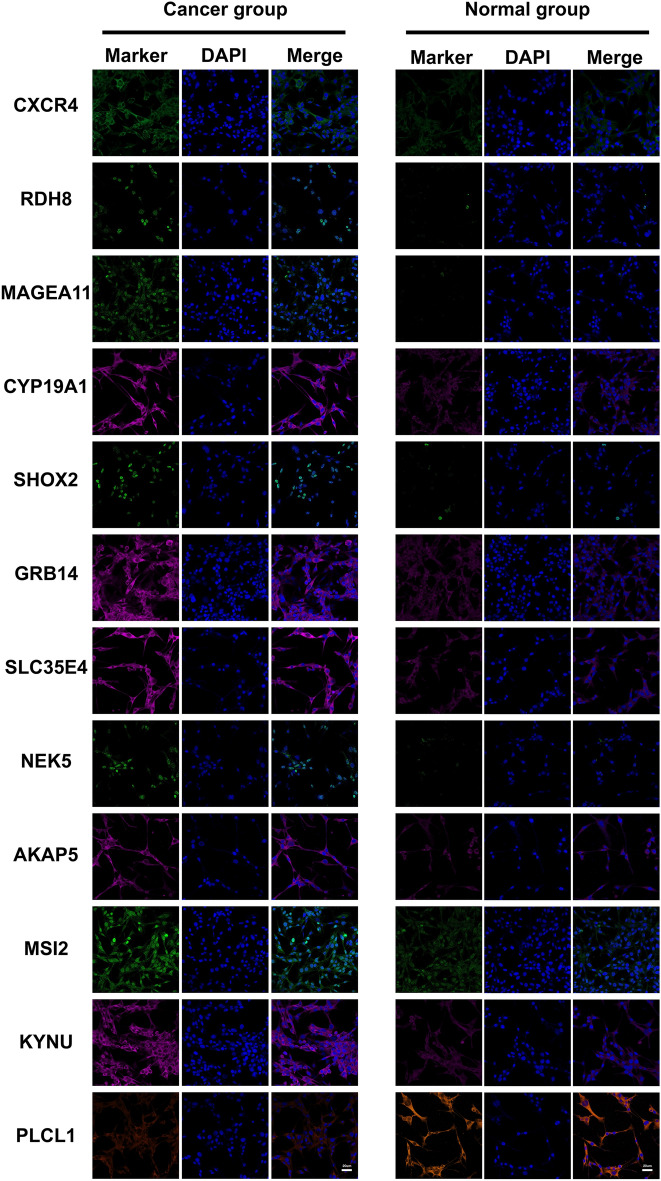
The expression level of proteins encoded by the signature genes detecting by immunofluorescence. The GES-1 cells were used as cancer group and AGS cells were used as control group. The nuclei were staining with DAPI. Scale bar, 20 μm.

The detailed information on the proteins encoded by the signature genes and their functions are showed in Supplementary Table 6, The mRNA expression analysis showed that *CXCR4, RDH8, MAGEA11, CYP19A1, SHOX2, GRB14, SLC35E4, NEK5, AKAP5, MSI2,* and *KYNU* were upregulated in the GC samples, whereas *PLCL1* was downregulated in GC patients (Fig. 3A). This result was validated in the GSE66229 cohort; analysis showed that *MAGEA11, CYP19A1, SHOX2, GRB14, SLC35E4, NEK5, AKAP5, MSI2*, and *KYNU* were upregulated in the GC samples while *PLCL1* was downregulated. Furthermore, there was a tendency for the upregulation of *RDH8* and *CYP19A1* in the GC samples; however, these changes were not statistically significant (Fig. 3B). Furthermore, we utilized qPCR and immunofluorescence to validate our findings, which demonstrated strong consistency with the bioinformatics analysis. (Figs. 4 and 5).

### Construction and assessment of the nomogram

**Figure 6:**
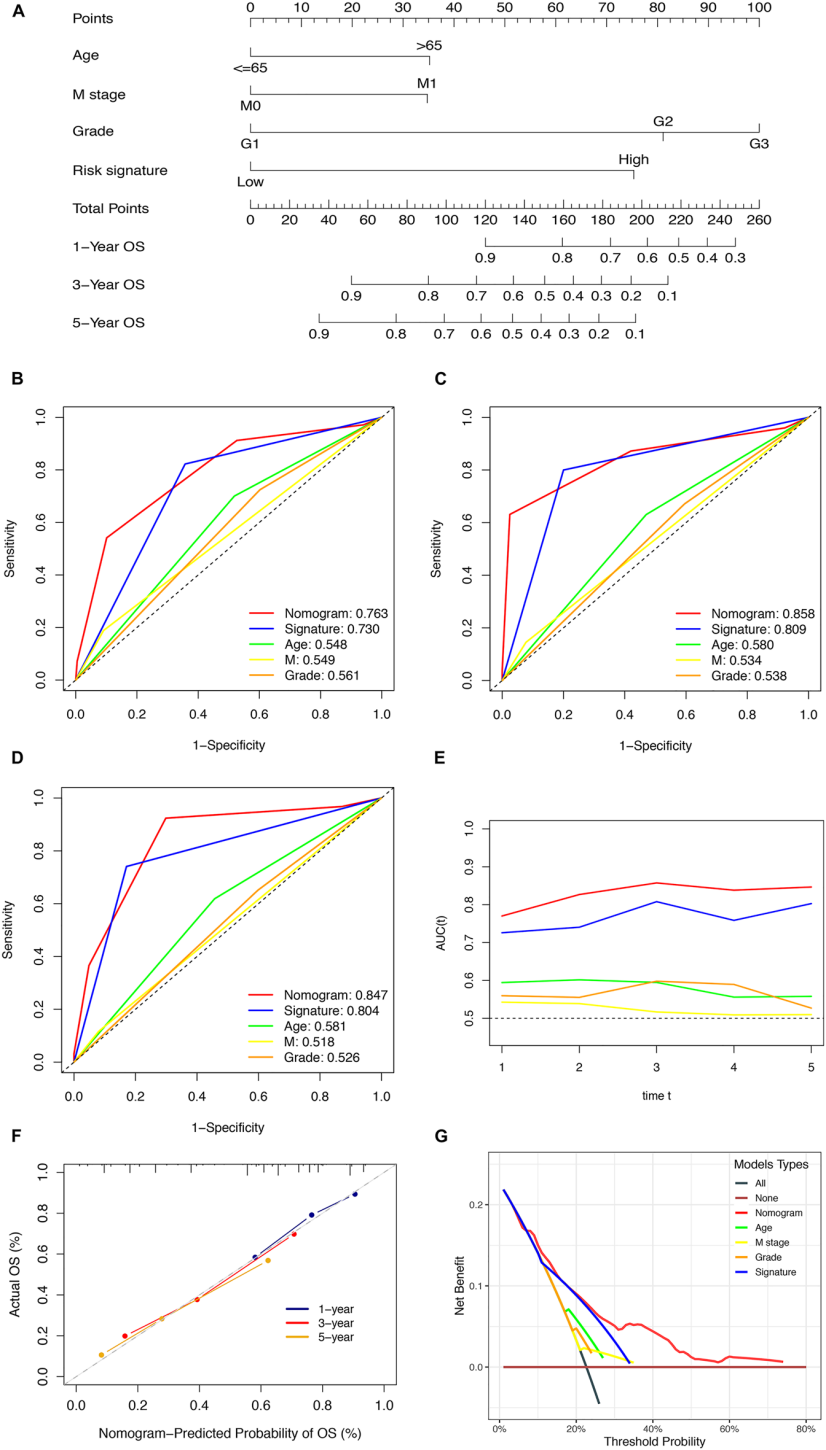
The establishment and assessment of nomogram. (**A**) The construction of the nomogram. (**B**–**D**) The AUC of the nomograms compared for 1-, 3-, and 5-year OS, respectively. (**E**) The time-dependent AUCs of the nomogram. (**F**) The calibration curve for assessing the agreement at 1-, 3-, and 5-year OS. (**G**) The DCA curves of the nomogram.

Next, we performed multivariate Cox regression analysis; analysis showed that the NKCAS as well as age, M stage, and tumor grade were all independent prognostic factors for GC patients (Table 1). Next, a nomogram was built by integrating clinical factors and the NKCAS risk model to predict the short- and long-term survival rates of patients with GC (Fig. 6A). The AUC values of the nomogram at 1-, 3-, and 5-years were 0.763, 0.858, and 0.847, respectively (Fig. 6B–D); these values remained higher than other factors over time, thus indicating that the nomogram had a good predictive performance in terms of prognosis (Fig. 6E). The calibration curve showed that the prediction values were highly consistent with the observation values (Fig. 6F). In addition, DCA found the nomogram more clinically valuable than the other factors (Fig. 6G). Collectively, these results demonstrated that the nomogram established based on the NKCAS risk signature along with clinical factors could be applied as a convenient tool to predict the prognosis of patients in clinical management.

**Table 1:** Multivariate Cox analysis for clinical variables.

Variables	HR (95% CI)	*P*-value
Age		
≤ 65	Reference	
> 65	2.07 (1.45–2.97)	< 0.001
Gender		
Female	Reference	
Male	1.28 (0.89–1.85)	0.181
Tumor grade		
G1-2	Reference	
G3-4	1.52 (1.05–2.21)	0.028
Pathological stage		
Stage I–II	Reference	
Stage III–IV	1.51 (0.89–2.54)	0.125
T		
T1-2	Reference	
T3-4	1.38 (0.84–2.28)	0.205
N		
N0	Reference	
N1-3	0.90 (0.51–1.59)	0.715
M		
M0	Reference	
M1	2.03 (1.22–3.38)	0.007
Risk score		
Low	Reference	
High	4.04 (2.74–5.95)	< 0.001

### Gene mutation atlas and tumor mutation burden analysis

**Figure 7:**
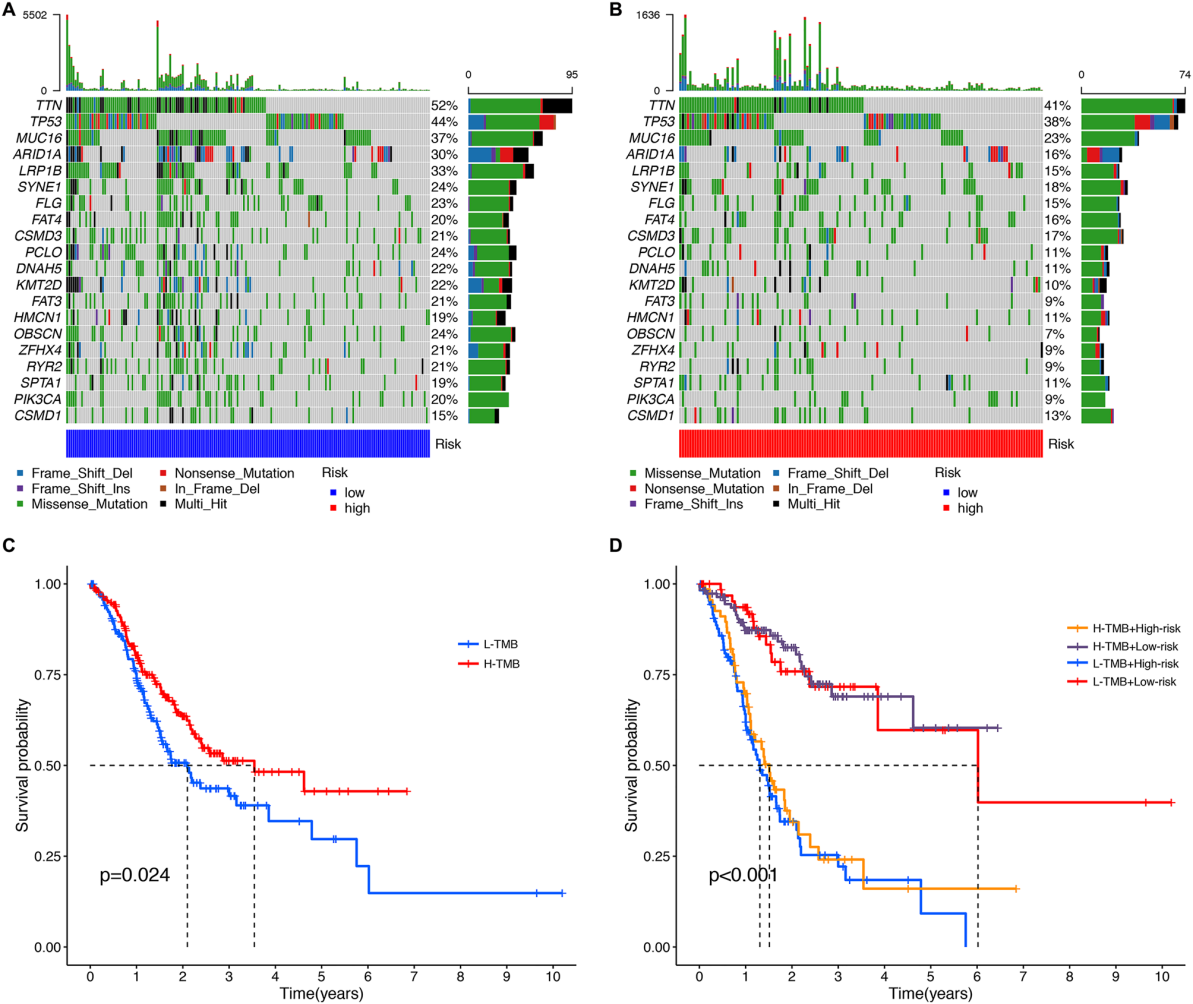
The gene mutational landscape. (**A**–**B**) Waterfall plot of the top 20 mutant genes in the low-, and high-risk group. (**C**) The Kaplan–Meier analysis curves for GC patients with low or high tumor mutation burden. (**D**) The Kaplan–Meier analysis curves for the GC patients stratified by NKCAS and TMB.

Summaries of gene mutations in different groups are shown in the Supplementary Figs. 3 and 4, respectively; analysis indicated that the LRG had a higher overall mutation frequency than the HRG. The top six genes with the highest mutation rates in the LRG were *TTN* (52%), *TP53* (44%), *MUC16* (37%), *LRP1B* (33%), *ARID1A* (30%), and *SYNE1* (24%); this was similar to the HRG with *TTN* (41%), *TP53* (38%), *MUC16* (23%), *SYNE1* (18%), *CSMD3* (17%), and *ARID1A* (16%). The most common type of mutation was missense mutation in both the LRG and the HRG (Fig. 7A–B). The results of tumor mutation burden analysis showed that a high TMB predicts a better prognosis only by not dividing GC patients between high and low risk (Fig. 7C). Moreover, we analyzed the impact of risk grouping combined with TMB on the survival outcomes of gastric cancer patients, considering the effect of risk groupings on patient prognosis; analysis showed that both the LRG and high TMB were favorable prognostic predictors for GC patients (Fig. 7D). Given this variation in prognosis, we subsequently executed an extensive analysis of the TME in GC.

### Characteristics of the TME in GC

**Figure 8:**
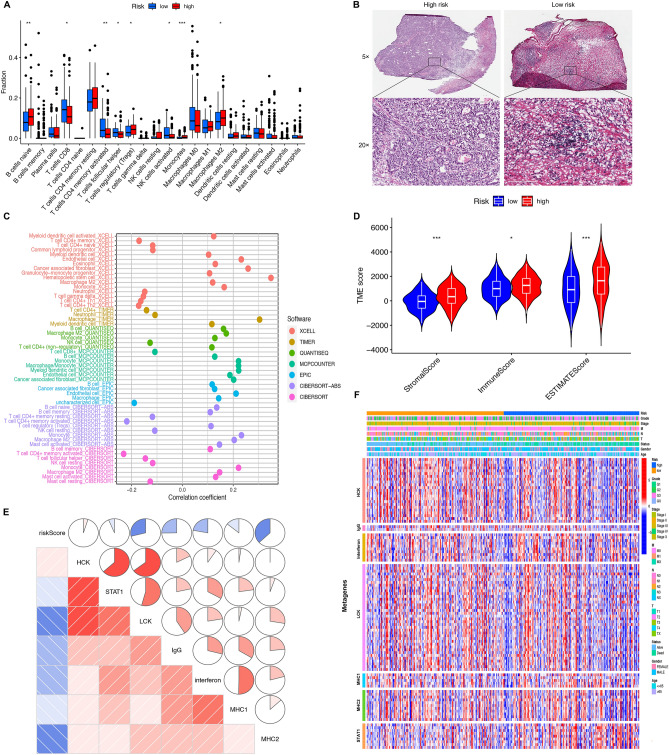
Landscape of tumor immune microenvironment. (**A**) Difference expression levels of 22 types of tumor-infiltrating immune cells between low-risk and high-risk groups. (**B**) H&E staining showed the infiltrated immune cells in TME. (Magnification, 5× & 20×.) (**C**) The relationship between the risk score and immune cell infiltration were validated in XCELL, TIMER, QUANTISEQ, MCPCOUNTER, and EPIC. (**D**) TME analysis based on ESTIMATE algorithm. (**E**) Correlogram was generated based on Pearson R-value between risk score and metagenes. (**F**) A Heatmap showed the relationship between risk score and inflammatory metagenes.

Analysis with the CIBERSORT algorithm analysis showed that the LRG had more infiltrating CD8+ T cells and CD4+ T cells, and more activated NK cells; The HRG had more infiltrating M2 macrophages, monocytes, and Tregs; these findings were consistent with data arising from the histopathological sections, thus indicating a higher immune killing ability in the LRG (Fig. 8A–B). Furthermore, the relationship between the risk scores and immune infiltrating cells were validated in XCELL, TIMER, QUANTISEQ, MCPCOUNTER, and EPIC; the results of these analyses were similar with those arising from CIBERSORT analysis (Fig. 8C). ESTIMATE analysis further suggested that the HRG group had a higher stromal score, immune score, and estimated score when compared with the LRG(Fig. 8D).

To identify the relationship between the NKCAS and inflammatory activities, we next investigated the association between the NKCAS and seven clusters of metagenes, specifically *HCK, LCK, IgG*, Interferon, *MHC-I, MHC-II, and STAT1*. Analysis showed that the LRG had higher expression levels of the 7 gene clusters (Fig. 8E–F), thus demonstrating higher levels of anti-tumor immunity.

### The NKCAS could predict the response to immunotherapy

**Figure 9:**
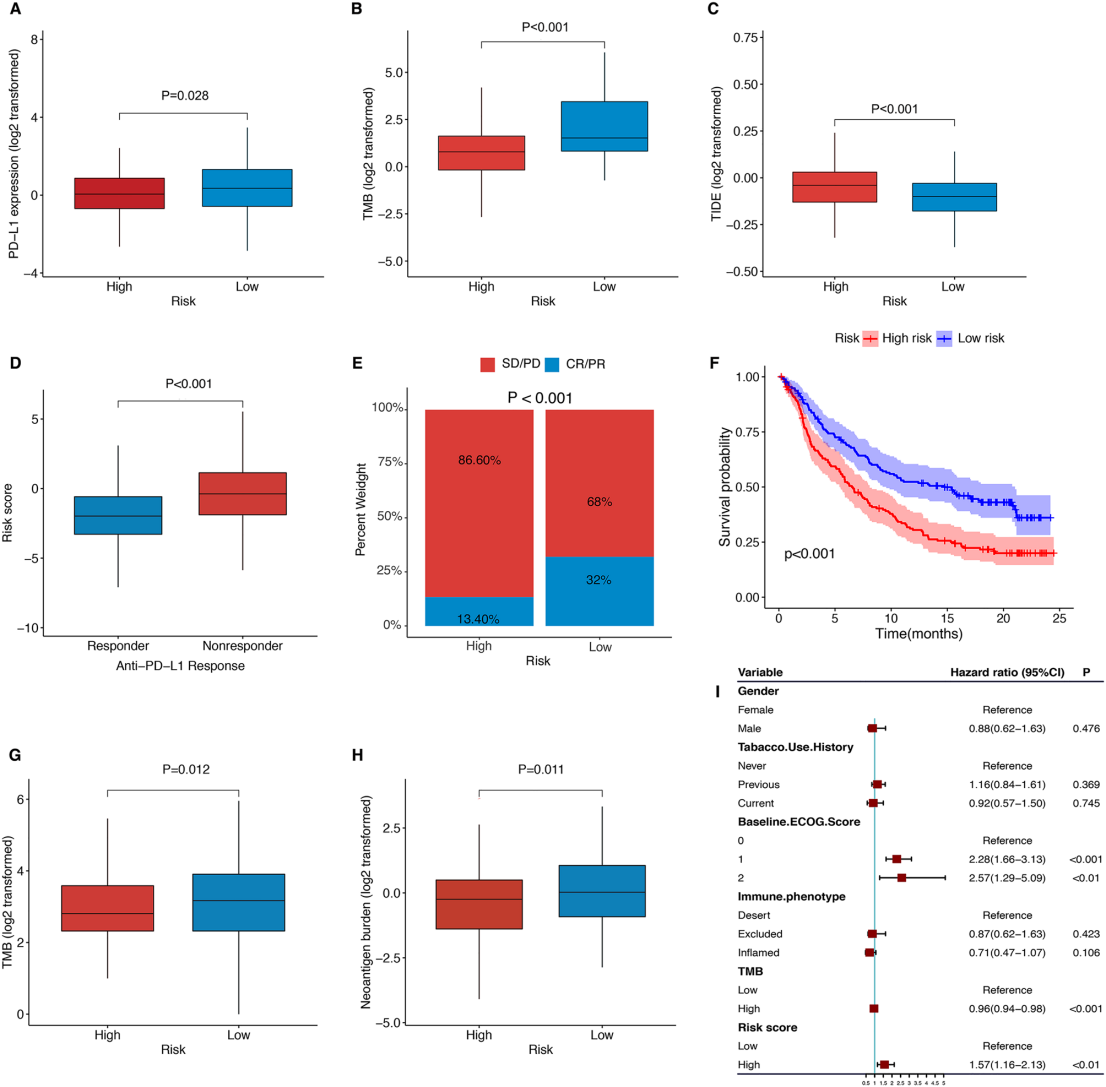
The role of NKCAS in predicting immunotherapy response. (**A**–**C**) Comparison of conventional immunotherapy predictors including PD-L1, TMB, and TIDE scores in low- and high-risk groups. (**D**) The relationship between the response to immunotherapy and the risk scores. (**E**) The proportion of response to immunotherapy in low- versus high-risk group. (**F**) Kaplan–Meier survival curve of the low- versus high-risk group in the immunotherapy cohort (IMvigor 210 cohort). (**G**–**H**) The TMB and neoantigen burden in low- versus high-risk group. (**I**) Multivariate Cox regression analysis of the NCMGS with features in the immunotherapy cohort.

Considering the important roles of NK cells in anti-tumor immunity, we next investigated whether the NKCAS could predict the response to immune checkpoint inhibitors in patients with GC. Analysis demonstrated that the LRG held higher levels of PD-L1 mRNA expression and TMB than the HRG but a lower TIDE score, thus indicating a greater response to treatment with ICBs (Fig. 9A–C). Furthermore, the IMvigor210cohort, featuring 348 patients taking anti-PD-L1 treatment, was included to further explore the prediction value of the NKCAS in immunotherapy response. Analysis suggested that lower risk scores were related to better objective responses (Fig. 9D). Anti-PD-L1 treatment was significantly more effective in the LRG than in the HRG (Fig. 9E). KM analysis further suggested that the LRG had better survival after receiving immunotherapy (Fig. 9F). In addition, the TMB and neoantigen burden were higher in the LRG, further indicating a better response to treatment with ICBs (Fig. 9G–H). The relationship between the NKCAS and survival in patients receiving immunotherapy persisted as statistically significant even when considering gender, smoking, ECOG score, immune phenotype, and TMB status (Fig. 9I). These results demonstrated that the NKCAS had a good predictive value for both immunotherapy and prognosis for GC patients.

## Discussion

With the ongoing advancement of scRNA-seq methods, it is now possible to study the cellular composition of malignant tumors at the level of single-cell resolution, and thus explore the heterogeneity of tumors and dissect the complex interactions between tumors and their immune microenvironment; this is crucial if we are to discover feasible therapeutic targets. Most existing studies have focused on the adaptive immunity generated by T cells; however, intrinsic immune cells have not received enough attention. NK cells are an important component of intrinsic immunity and play an important role in anti-infection and tumor-killing processes. Several studies have reported on the significant role of NK cells in GC. For example, previous study reported that NK cells were independent prognostic factor for GC^20^. The number of NK cells decreased in advanced-stage GC, and their density could predict favorable survival outcomes. Mechanistically, tumor-derived PGE2 can induce NK cell dysfunction in GC, hindering their anti-tumor activity and promoting tumor growth and progression^21^. Given the importance of NK cells in gastric cancer. Here, we aimed to identify the NK cell signature genes of GC via scRNA-seq analysis. Then, we developed a novel NKCAS for the prognosis of GC in the TCGA cohort. The GEO cohort was used to further verify the predictive performance of the NKCAS. In addition, the LRG had a greater number of infiltrating immune cells, gene mutations, and exhibited a stronger response to treatment with ICIs. Previous study have reported the predictive value of NK signatures in gastrointestinal cancer. This NK signature consists of SLC2A3 and POU2F2, can predict both the prognosis of colon cancer patients and the efficacy of immunotherapy^22^. However, this study has relatively little research on the relationship between NK signatures and the tumor microenvironment and gene mutations. Overall, the signatures of natural killer cells (NK) in gastric cancer are largely unknown. Therefore, in the current study, we examined the prognostic value of the NK signature, as well as its association with the tumor microenvironment, gene mutations, and response to immunotherapy.

In the current study, the NKCAS was established with 12 NK cell signature genes including *CXCR4, RDH8, MAGEA11, CYP19A1, SHOX2, GRB14, SLC35E4, NEK5, AKAP5, MSI2, KYNU*, and *PLCL1*. Some of these genes had previously been reported to play an important role in cancer. *CXCR4* encodes a CXC chemokines receptor specific for stromal cell-derived factor-1 and it can combine with CXCL12 to facilitate NK cell development in adults^23^. Besides, *CXCR4* has been reported to be overexpressed in numerous malignancies and is associated with tumor growth, invasion, angiogenesis, metastasis, recurrence, and drug resistance^24^. The upregulation of *CXCR4* promoted the invasion and migration of GC cells by inducing epithelial-mesenchymal transition (EMT), thus weakening the prognosis of patients^25^. *MAGEA11* was previously found to be highly expressed in breast cancer, bladder cancer, and laryngeal squamous cell carcinoma^26–28^; the positive expression of this gene was associated with the progression of malignant tumors, thus leading to poor survival^29,30^. *CYP19A1*, a member of the cytochrome P450 family, was previously found to be highly expressed in GC and associated with an adverse prognosis; the silencing of its expression could be useful for GC treatment^31,32^. A previous study showed that *SHOX2* methylation represent a potential biomarker for some cancers^33–37^. This gene was also shown to be related to unfavorable distant metastasis-free survival and could promote *WASF3* transcriptional activity to induce the growth and metastasis of breast cancer. *GRB14* was previously shown to be a poor prognostic predictor for colorectal cancer; the overexpression of this gene can enhance cell invasion and result in the metastases of thyroid cancer^38,39^. Interestingly, *GRB14* was identified as a good prognostic factor for breast cancer patients; the overexpression of *GRB14* was shown to inhibit estrogen-induced cell cycle progression^40,41^. *NEK5* activity is known to regulated the mesenchymal and phenotype of breast cancer cells and can promote cell proliferation via the up-regulation of Cyclin A2^42,43^. *MSI2* has the potential to be a novel therapeutic target for cancers^44^ and can promote cancer progression and drug resistance via multiple signaling pathways^45–49^. KYNU encodes kynureninase, an enzyme that catalyzes the cleavage of L-kynurenine, which has been found to block cytokine-mediated up-regulation of the expression and function of NKp46 and NKG2D, thereby inducing NK cell-mediated killing^50^. In addition, high expression levels of *KYNU* were previously shown to be related to poor disease-free survival in cancer patients^51^. It has been reported that the depletion of *KYNU* could inhibit the growth of cancer cells via the PI3K/AKT signaling pathway^52^. A previous study showed that the upregulation of *PLCL1*, mediated by the overexpression of *CHD5*, could suppress the invasion and migration of neuroblastoma cells^53^. In addition, lipid browning mediated by PLCL/UCP1 promotes tumor cell “slimming” and reduces abnormal lipid accumulation, thus repressing the progression of clear cell renal cell carcinoma^54^. In particular, there is limited information available regarding the involvement of *SLC35E4, AKAP5*, and *RDH8* in malignancies; only a few bioinformatics studies have addressed their potential as prognostic predictors for cancer. Consequently, further research is now required to fully understand their biological roles. In addition, we analyzed the expression levels of marker genes and validated these results using independent external datasets. Due to the importance of the signature in the prognosis, TME, and immunotherapy of GC. The signature's included genes may represent potential molecular mechanisms for GC.

The predictive ability of the NKCAS was further evaluated in the GEO dataset. Analysis showed a good consistency in all datasets, thus demonstrating a strong robustness and repeatability. Moreover, we developed a nomogram to predict the survival probabilities of GC patients. The outcomes of multiple methods (AUCs and calibration curves) indicated a good predictive performance of the nomogram. Furthermore, DCA analysis suggested that applying this nomogram into clinical management might provide more net-benefit for GC patients. The nomogram could improve guidance on patient prognosis and facilitate the efficient utilization of medical resources.

The TME plays an important role in tumor progression and antitumor response^55^. Here, we investigated the characteristics of the GC TME in distinct risk groups. The stromal, immune, and ESTIMATE scores were lower in the LRG than in the HRG. This indicated a higher proportion of stromal cells and lower tumor purity in the HRG. It has been reported that stromal cells related to the TME can promote tumor growth and hinder immunity, and that low tumor purity is associated with an unfavorable prognosis and immune-evasion phenotype. Thus, stromal changes during the development of GC may be detrimental^56,57^. As a crucial element of the TME, the allocation of immune infiltrating cells also fluctuates among different risk categories. CIBERSORT results showed that tumors from the LRG had a high infiltration of CD4+ T cells, CD8+ T cells, NK cells, and neutrophils. Previous research indicated that a significant abundance of T cells may denote a “hot” tumor phenotype, which can improve the host's anti-tumor defenses and enhance the efficacy of immunotherapies^58^. This ultimately leads to improved overall survival rates for GC patients^59^. NK cells are a crucial facet of innate immunity and play a significant role in organizing antitumor immune responses. A previous study showed that the high infiltrations of NK cells in solid tumors was associated with a favorable prognosis^10^. The CIBERSORT results also suggested that tumors from the HRG had a high infiltration of monocytes, B cells, Tregs, and M2 macrophages. The promotion of tumor growth and invasion may be facilitated by anti-inflammatory M2 macrophages. Inflammatory monocytes, on the other hand, may promote the extravasation of tumor cells, thus facilitating cancer metastasis. Additionally, a study has suggested that patients with GC who have high peritumoral TIGIT + CD20 + B cell infiltration may experience inferior clinical outcomes due to the effect of these cells on the exhaustion of CD8+ T cells. Furthermore, it has been reported that Foxp3+ Tregs are highly expressed in gastric cancer and are associated with poor clinical outcomes. This may explain the clustering of M2 macrophages, B cells, monocytes, and Tregs in the HRG^60–63^. Collectively, these results indicated that low-risk patients may have a relatively active anti-tumor immune response.

Furthermore, we investigated the relationship between the risk groups and the immune-related metagene, which reflects various inflammatory and immune activities, as previously reported^19^. We found that the risk score was negatively related to *LCK, STAT1*, interferon, *IgG, MHC-I*, and *MHC-II* clusters. *LCK*, a Src-related protein tyrosine kinase, is essential for the development and activation of T cells^64^. Activated *LCK* signaling can potentiate CD8+ T cell activation and anti-tumor responses thereby improving survival^65^. *STAT1* inhibits T cell exhaustion and myeloid derived suppressor cell accumulation; thus, the selective induction of *STAT1* phosphorylation in cancer patients could potentially improve antitumor immune responses^66^. *MHC* molecules are closely associated with immune response and immune regulation, and tumors can circumvent T cell-mediated cytotoxic responses via the loss of *MHC*^67^. Given this, the therapeutic increase *MHC* expression could sensitizes cancer cells to T cell-dependent killing, thus increasing the efficacy of immune checkpoint blockade^68^. Interferons exert a synergistic effect on anti-tumor immunity, and can active *MHC I* to enhance protective anti-tumor CD8+ T cell immunity^69,70^. Collectively, our data show that the high-risk patients exhibit low inflammatory activity and immune activity; this may explain their poorer prognosis, at least in part.

Given the differences in the TME between risk groups, we next investigated the potential usefulness of the NKCAS in predicting the response to immunotherapy. We evaluated the relationship between the NKCAS and currently recognized markers such as *PD-L1* expression, TMB, and TIDE score. Analysis showed that *PD-L1* expression and TMB were significantly higher but TIDE score was lower in the LRG, thus indicating that low-risk tumors are more immunogenic and may respond better to immunotherapy. Collectively, these results revealed a potential power of NKCAS for predicting immunotherapy response. Thus, we used the immunotherapy cohort (IMvigor210) to further verify this hypothesis. Particularly, the IMvigor210 cohort was about uroepithelial cancer receiving immunotherapy. Multiple previous studies have confirmed the use of this dataset in other cancer types to validate the predictive signatures developed. Our result suggested that low-risk patients had a higher neoantigen burden and TMB, thus demonstrating higher immunogenicity. Consequently, these patients were more sensitive to anti-PD-L1 treatment thereby achieving a better prognosis. In summary, patients in the LRG were more likely to benefit from immunotherapy. With more in-depth evaluation, the NKCAS might become a reliable biomarker for immunotherapy response.

The strength of this study is that we initially created a durable signature (NKCAS) by combining scRNA-seq and bulk RNA-seq. The signature was subsequently verified with an external dataset and exhibited good predictive ability for GC prognosis. Furthermore, we explored the relationship between NKCAS and clinicopathological factors, the tumor immune environment, and immunotherapy response; this provides insight into the precise immune characteristics that underlie NKCAS and may be critical for individuals with GC. Regardless of these strengths, our study has limitations that should be acknowledged. First, the signature was built using data from public datasets. Further validations are now needed by research undertaken in multiple centers and in a prospective clinical cohort. Second, we did not perform any in vivo or in vitro experiments in the current study; further functional experiments on the signature are required to verify our silico results.

In conclusion, in this study, we developed and validated a novel prognostic signature consisting of 12 NK cell marker genes by comprehensively analyzing scRNA-seq and bulk RNA-seq. Our gene signature could serve as a powerful biomarker and may be able to predict prognosis and immunotherapy response in GC patients. Our study provides new insights into the role of immune cell marker genes in the prognosis and immunotherapeutic response of GC patients.

### Supplementary Information

Supplementary Information.

## Data Availability

The data that support this study are openly available in online repositories including the TCGA database (https://portal.gdc.cancer.gov/repository), GEO database (https://www.ncbi.nlm.nih.gov/geo/), and IMvigor210 cohort (http://research-pub.gene.com/IMvigor210CoreBiologies).

## Abbreviations

GC: Gastric cancer
NKCAS: NK cell-associated signature
scRNA-seq: Single-cell RNA-sequencing
ICB: Immune checkpoint blockade
TME: Tumor microenvironment
TCGA: The cancer genome atlas
GEO: Gene expression omnibus
PCA: Principal component analysis
t-SNE: T-distributed stochastic neighbor embedding
LASSO: The least absolute shrinkage and selection operator
ROC: The receiver operating characteristic
AUC: The area under curve
DCA: Decision curve analysis
LRG: Low-risk group
HRG: High-risk group
TIDE: Tumor immune dysfunction and exclusion
TMB: Tumor mutation burden
