# Supplementary material for: Integrated analysis of single-cell and bulk RNA-sequencing reveals a novel signature based on NK cell marker genes to predict prognosis and immunotherapy response in gastric cancer
Source: Sci Rep. 2024 Apr 1;14:7648. doi: 10.1038/s41598-024-57714-7 (PMC10985121; doi:10.1038/s41598-024-57714-7)
Supplement: Supplementary file 1 — Supplementary Information. [file 41598_2024_57714_MOESM1_ESM.pdf]

Integrated analysis of single-cell and bulk RNA-sequencing reveals a novel signature based on NK cell marker genes to predict prognosis and immunotherapy response in gastric cancer

Supplementary Figure 1: NK cell marker genes associated with the GC patients' OS.

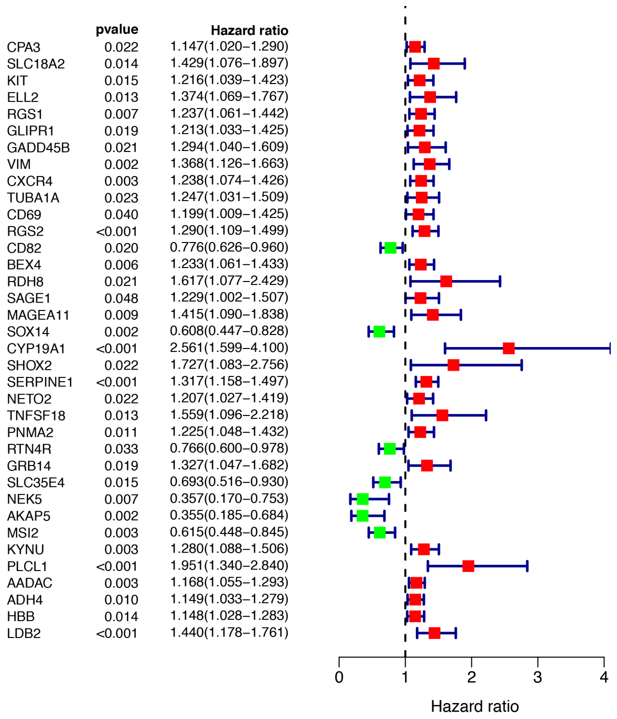

Supplementary Figure 2: The distribution of risk score, survival status (**A-B**), and expression of signature genes (**C-D**) in both TCGA and GEO cohorts.

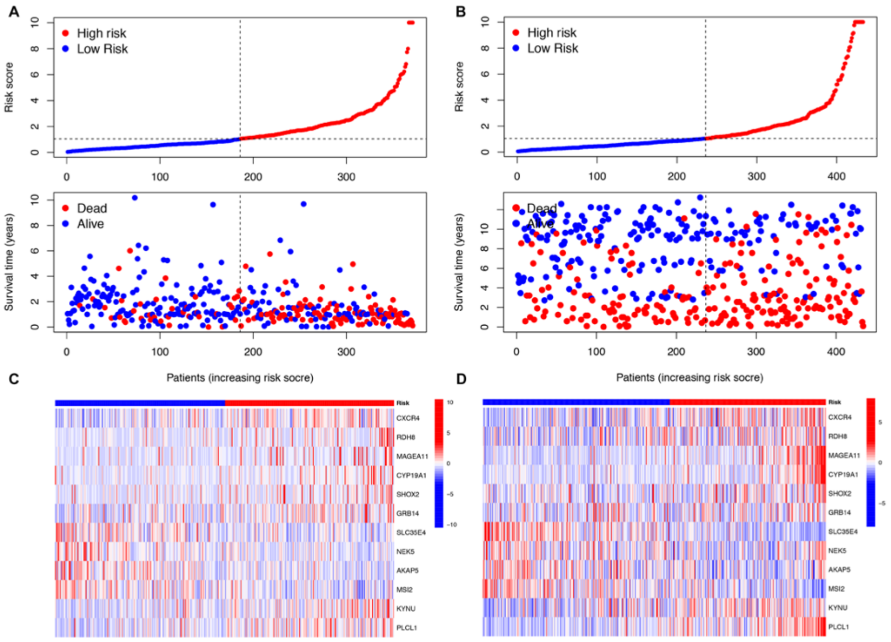

Supplementary Figure 3: Summaries of gene mutation profiles for low-risk group.

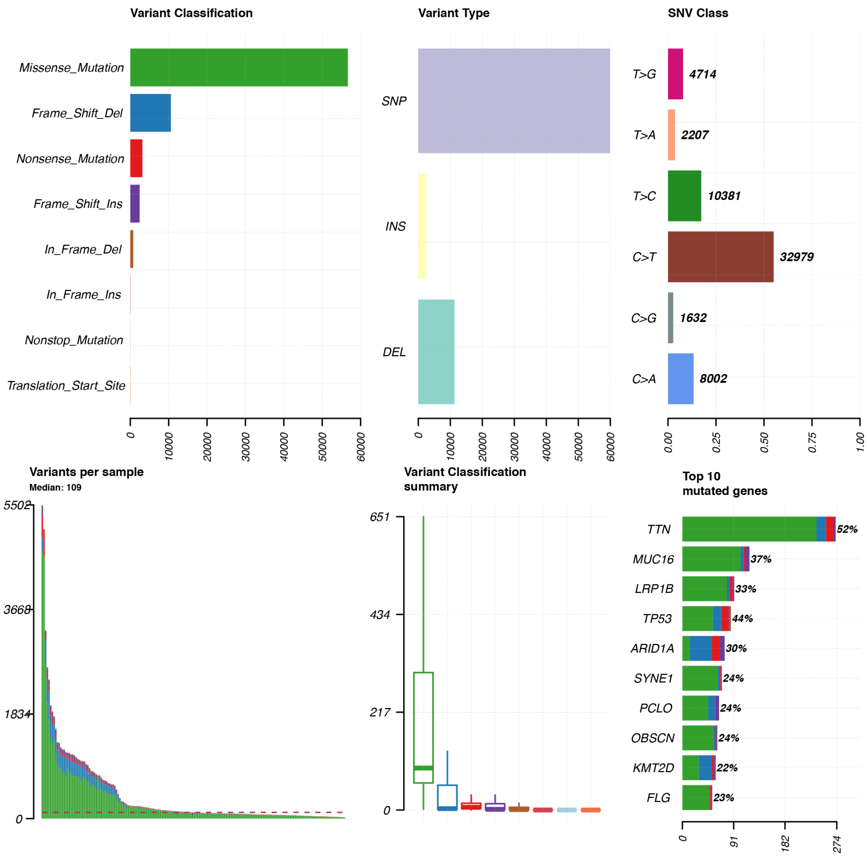

Supplementary Figure 4: Summaries of gene mutation profiles for high-risk group.

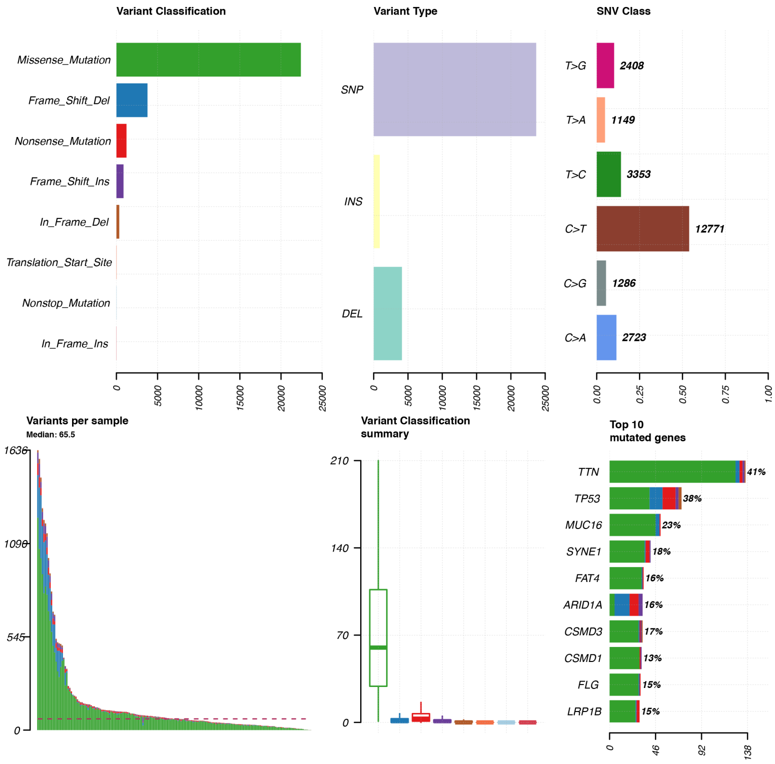

Supplementary Table 1: The baseline features of GC patients in TCGA and GEO datasets.

**Features**	**TCGA (n=371)**	**GSE84437 (n=433)**
Age, years (mean±SD)	65.54±10.55	60.06±11.58
Gender		
Male	230	296
Female	141	137
Tumor grade		
G1	25	—
G2	129	—
G3	209	—
G4	8	—
Pathologic stage		
I-II	173	157
III-IV	198	276
Tumor size		
T1	16	11

T2	70	38
T3	161	92
T4	124	292
Lymph node metastasis		
N0	109	80
N1	115	188
N2	72	132
N3	75	33
Metastasis status		
M0	319	347
M1	52	86

The “－” indicates that the value is not available; HR, hazard ratio; CI, confidence interval.

Supplementary Table 2. The baseline features of patients in IMvigor210 cohort.

**Features**	**n=348**
OS, months (mean±SD)	10.25±7.65
Survival status	
Alive	232
Dead	116
Gender	
Female	76
Male	272
Tobacco. Use. History	209
Current	35
Never	116
Previous	197
Baseline. ECOG. Score	198
0	134
1	196
2	18

Immune. Phenotype	161
Desert	76
Exclude	134
Inflamed	74
Unknown	64
Binary Response	72
CR/PR	68
SD/PD	230
Unknown	50
Mutation burden per MB (mean±SD)	10.86±9.57

OS, overall survival; *ECOG, eastern cooperative oncology group;* CR, complete remission; PR, partial remission; SD, stable disease; PD, progressive disease.

Supplementary Table 3: The primers sequences of mRNAs.

**Gene name**	**Forward sequence(5'-3')**	**Reverse sequence(5'- 3')**
CXCR4	ACTACACCGAGGAAATGGGCT	CCCACAATGCCAGTTAAGAAGA
RDH8	TCAGGAATTGGTCTGGAACTTGC	CAGCACGTCCACTTCTCCC
MAGEA11	TCCCAGGATCTGCCAAGAGTC	CCCCACAGCACTTGTTCTC
CYP19A1	TGGAAATGCTGAACCCGATAC	AATTCCCATGCAGTAGCCAGG
SHOX2	CAAAGAGGATGCGAAAGGGAT	AGTGGGTCTCGTCAAAAAGCC
GRB14	AACCCTTTTCCTGAGCTATGC	TCACTGGGTACATCTAAAGCCC
SLC35E4	TGCCGAGTCCTACTGCTCA	TGGTAGTAACCAGTTGTGCCA
NEK5	GCAAGCACTGTGTCATAAAAGAG	CACCCCGTTGTCTATTGATCC
AKAP5	GGCCTCACTCAAACGTCTTGT	GGTTGCATTTCACCCTCCAAT
MSI2	ACCTCACCAGATAGCCTTAGAG	AGCGTTTCGTAGTGGGATCTC
KYNU	GGCTCTCCACCTAGATGAGGA	GCTGCTATTTTGGCCCACTTAT
PLCL1	AAAGTCCGGCCAAATTCTCG	TTTCCGTGTTTTTCCCCAGTC
GAPDH	TGCACCACCAACTGCTTAGC	ATCGAGTGAAGGACCTGGC

Supplementary Table 4: The signature genes of NK cells.

TPSB2	VAPA	BTK	SOD2	TSC22D1	ANXA2	TXK
TPSAB1	GYPC	SRGN	FABP5	CORO1A	BIRC3	NCAM1
CPA3	LCK	CALB2	CTSS	PLIN2	NSMCE1	CD247
HPGDS	YPEL5	BACE2	IGKC	HLA-DRB1	ARHGAP18	KLRB1
MS4A2	RGS1	H3F3B	CTSZ	TNFSF10	HLA-DQB1	CTSW
CTSG	HSD17B12	FTH1	IGLC2	HLA-DPB1	PPP1R15A	KRT86
GATA2	TRBC1	SMYD3	CCL4	ASAH1	HLA-DRA	MATK
HPGD	GLIPR1	SVOPL	SERPINA1	PRDX6	CCL5	CD160
RGS13	IL7R	VIM	S100A9	GALC	DDIT4	LDB2
C1orf186	GPR183	MITF	IGHA1	CD3D	ITGB2	GZMK
VWA5A	HLA-DMA	SLC45A3	IGHG3	BHLHE40	RDH8	TYROBP
LTC4S	KDM6B	STXBP6	C1QB	RGCC	SAGE1	CST7
SLC18A2	AHR	CSF1	IGLC3	APOC1	MAGEA11	IFITM1
CLU	EZR	GRAP2	IGHG1	CTSB	SOX14	SAMD3
NFKBIA	ACAP1	HLA-B	C1QA	APOE	CYP19A1	SH2D1A
LAPTM4A	EML4	HINT1	IGHG4	CYTIP	SHOX2	PRF1
CD9	CTSH	ACTG1	LYZ	ID2	SERPINE1	PVRIG
KIT	GZMA	ZFP36L2	STX3	EVL	NETO2	TARP
FCER1A	TUBA4A	CD74	HLA-A	RGS10	TNFSF18	APOBEC3G
DUSP6	CD7	IL32	RENBP	PTGS2	PNMA2	RAC2
LMNA	PTPRC	CXCR4	CD69	NFKBIZ	RTN4R	GLIPR2
MAOB	STK17A	HES1	LEO1	ITM2C	GRB14	FGR
IL1RL1	S100A10	CD3G	ACOT7	HLA-DPA1	SLC35E4	GNLY
ANXA1	IL2RG	DNAJB1	CLIC1	CPM	NEK5	PTPRCAP
LMO4	ARL4C	ARID5B	SELK	RGS2	AKAP5	LCP1
HDC	SPOCK2	RORA	LSP1	CTNNBL1	MSI2	PTPN22
GLUL	GADD45B	LIMD2	STMN1	LTB	KYNU	GIMAP7
CD63	SAMSN1	TWISTNB	HLA-C	PEBP1	PLCL1	PRKCH
CAPG	ADRB2	CLEC2D	MSRA	CD2	AADAC	HCST
GCSAML	FOSB	TUBA1A	GPR65	CD82	ADH4	RPS27
ACSL4	PTGS1	HLA-DMB	ANKRD28	BEX4	HBB	IPCEF1
Gene	TMEM233	NKG7	BTG1	ISG20	KLRC1	TMSB4X
RAB32	FCER1G	SGK1	SDCBP	CD3E	SH2D1B	ALOX5AP
ELL2	LAT2	HOPX	TMEM176B	ALOX5	IL2RB	SLC2A3
HLA-DQA1	CNRIP1	DUSP4	ARHGEF6	TRBC2	KLRF1	PREX1
DDAH2	RAB27B	COTL1	TRAC	FYB	SIGLEC17P	DOCK10
AOAH	SH3BGRL3	JUN	TNFRSF4	SH2D2A	SAT1	
GZMB	PPP1R12A	IQGAP1	IER3	CALM1	LST1	
IFITM2	TMA7	RPS8	CCL3	CELF2	GRN	
WIPF1	IER2	RPL6	TTN	RPL7A	LAIR2	

ETS1	RPLP1	NR4A2	EVI2B	RPS3A	NAMPT
DOK2	RPS23	FAU	CD96	CRIP1	CD68
ARHGDIB	EOMES	DDX5	RPS11	RPL13	BCL2A1
ADGRG1	STK17B	GNG2	JUNB	GATA3	YWHAH
LBH	RPL19	RPS24	RPL23A	RPL3	CSTB
SLFN5	MYL12A	NEAT1	TMSB10	PLEK	BATF
RPS15A	CD52	TXNIP	CYTH4	RPL35A	
CLEC2B	RPS9	ABCB1	CNN2	JUND	
GSTP1	RPS3	DUSP1	FYN	RPS2	
RPS27A	TPT1	RPL30	CCND2	RPS4X	
DUSP2	RPL18A	RPS25	RPLP2	HSPA1A	
AKNA	RPL34	TPSD1	FGD3	PLPP1	
PYHIN1	PABPC1	TRDC	RPL18	WASHC1	
RUNX3	RPS19	RHEX	KLRD1	HERPUD1	
RPL41	FOS	AL157895.1	RPS18	SOCS3	
TC2N	MS4A1	KIR2DL4	B2M	GLRX	
RPS29	RPL26	TMIGD2	RPS15	CD27	
RPS14	RPSA	LINC01871	RPL10	CORO1B	
PFN1	RPS6	AREG	GMFG	AIF1	

Supplementary Table 5: Genes determined by LASSO analysis.

Gene	Coef
SLC18A2	0.031
RGS1	0.012
CXCR4	0.052
CD82	-0.028
RDH8	0.207
SAGE1	0.043
MAGEA11	0.217
SOX14	-0.200
CYP19A1	0.442
SHOX2	0.064
SERPINE1	0.152
NETO2	0.108
TNFSF18	0.001
PNMA2	0.038
RTN4R	-0.040
GRB14	0.127
SLC35E4	-0.153

NEK5	-0.392
AKAP5	-1.040
MSI2	-0.123
KYNU	0.149
PLCL1	0.355
AADAC	0.041
ADH4	0.076
HBB	0.033

Supplementary Table 6: The proteins encoded by the signature genes and their functions.

CXCR4	CXCR4 encodes a CXC chemokine receptor specific for stromal cell-derived factor-1. It can combine with CXCL12 to facilitate NK-cell development in adults.
RDH8	RDH8 encodes a member of the short-chain dehydrogenase/reductase family. The encoded protein catalyzes the reduction of all-trans-retinal to all-trans-retinol, the first reaction step of the rhodopsin regeneration pathway.
MAGEA11	MAGEA11 is a cancer/testis antigen that belongs to the type I MAGE family of proteins. Research studies have demonstrated that MAGE-A11 broadly functions as an oncogene by complexing with the HUWE1 E3 ubiquitin ligase. This molecular complex promotes the aberrant ubiquitin-dependent proteasomal degradation of PCF11 and subsequent dysregulation of 3' UTR processing of mRNA transcripts encoding core components of oncogenic and tumor suppressor signaling pathways.
CYP19A1	CYP19A1 encodes a member of the cytochrome P450 superfamily of enzymes. The cytochrome P450 proteins are monooxygenases which catalyze many reactions involved in drug metabolism and synthesis of cholesterol, steroids and other lipids.
SHOX2	This gene is a member of the homeobox family of genes that encode proteins containing a 60-amino acid residue motif that represents a DNA binding domain. It is a regulator of cell proliferation and apoptosis and an inducer of EMT. In addition to its functions in the progression of cancer, SHOX2 is also critical for skeletal development, embryonic development, and cardiovascular system differentiation.
GRB14	GRB14 encodes a growth factor receptor-binding protein that interacts with insulin receptors and insulin-like growth-factor receptors. This protein likely has an

	inhibitory effect on receptor tyrosine kinase signaling and, in particular, on insulin receptor signaling.
SLC35E4	The function of the SLC35E4-encoded protein is currently unknown.
NEK5	NEK5 encodes a protein kinase that belongs to the Nima-Related Kineses (NEKs) family. In this NEK family, 11 genes are encoding different serine/threonine kinases, which catalytic domains have 40-45% amino acid sequence identity with NIMA's (Never In Mitosis A-related kinases) catalytic domain. It affects tumor progression by modulating the cell cycle, influencing DNA synthesis and repair, and regulating intercellular mesenchymal transition.
AKAP5	AKAP5 encodes a member of the AKAP family. The encoded protein binds to the regulatory subnit of PKAIIβ, anchoring the enzyme to the plasma membrane and sites of cytoskeletal/membrane junctions. The other binding domains of AKAP5 have been shown to interact with calmodulin, PP2B, and calcineurin suggesting that AKAP5 may act to coordinate the calcineurin suggesting the AKAP5 may act to coordinate the cAMP- and Ca2+-sensing pathways in various cell types.
MSI2	MSI2 encodes an RNA-binding protein that is a member of the Musashi protein family. The encoded protein is transcriptional regulator that targets genes involved in development and cell cycle regulation.
KYNU	KYNU codes for Kynureninase, an enzyme that catalyzes the cleavage of L-kynurenine, a tryptophan catabolite which has been found to prevent the cytokine-mediated up-regulation of the expression and function of NKp46 and NKG2D, two receptors responsible for the induction of NK-cell-mediated killing.
PLCL1	The function of the PLCL1-encoded protein is less unknown. Study showed that PLCL1 may affect tumor progression through modulation of autophagy, induction of apoptosis, and regulation of lipid metabolism.
